# Atmospheric formaldehyde production on early Mars leading to a potential formation of bio-important molecules

**DOI:** 10.1038/s41598-024-52718-9

**Published:** 2024-02-09

**Authors:** Shungo Koyama, Arihiro Kamada, Yoshihiro Furukawa, Naoki Terada, Yuki Nakamura, Tatsuya Yoshida, Takeshi Kuroda, Ann Carine Vandaele

**Affiliations:** 1 Graduate School of Science, Tohoku University, Sendai, Miyagi 980-8578 Japan; 2 Graduate School of Science, The University of Tokyo, Tokyo, Japan; 3 Division for the Establishment of Frontier Sciences of Organization for Advanced Studies, Tohoku University, Sendai, Japan; 4 Royal Belgian Institute for Space Aeronomy, BIRA-IASB, Brussels, Belgium

**Keywords:** Astrobiology, Atmospheric chemistry

## Abstract

Formaldehyde (H_2_CO) is a critical precursor for the abiotic formation of biomolecules, including amino acids and sugars, which are the building blocks of proteins and RNA. Geomorphological and geochemical evidence on Mars indicates a temperate environment compatible with the existence of surface liquid water during its early history at 3.8–3.6 billion years ago (Ga), which was maintained by the warming effect of reducing gases, such as H_2_. However, it remains uncertain whether such a temperate and weakly reducing surface environment on early Mars was suitable for producing H_2_CO. In this study, we investigated the atmospheric production of H_2_CO on early Mars using a 1-D photochemical model assuming a thick CO_2_-dominated atmosphere with H_2_ and CO. Our results show that a continuous supply of atmospheric H_2_CO can be used to form various organic compounds, including amino acids and sugars. This could be a possible origin for the organic matter observed on the Martian surface. Given the previously reported conversion rate from H_2_CO into ribose, the calculated H_2_CO deposition flux suggests a continuous supply of bio-important sugars on early Mars, particularly during the Noachian and early Hesperian periods.

## Introduction

Present-day Mars is extremely cold and dry, but many geomorphological and geochemical evidence, such as valley networks, suggest an active water cycle at 3.8–3.6 Ga^1^. The detection of phyllosilicates over the Noachian terrain also supports the existence of widespread liquid water on early Mars^2^. The habitability of Mars has been of great interest that triggered previous and ongoing Martian explorations. Although water is a probable requirement of Martian habitability, this molecule is not an organic compound in genomic and catalytic bio-molecules that supports the fundamentals of life. Investigations on the organic synthesis on ancient Mars fill the gap by verifying the possibility, environment, and age of the chemical evolution to potential ancient Martian life.

Formaldehyde (H_2_CO) is simple organic matter that can be formed through various chemical reactions in planetary atmospheres. H_2_CO is a highly soluble and reactive molecule, and thus has the potential to play a significant role in the abiotic formation of bioorganic molecules^3^. For example, amino acids are formed by reactions involving H_2_CO, NH_3_, and HCN via the Strecker reaction^4^. Additionally, in ammonia-involving formose-type reactions, the condensation of H_2_CO with NH_3_ yields various amino acids^5,6^. The formose reaction is a thermally driven aqueous process that generates many sugars from H_2_CO, including ribose, a fundamental building block of RNA that is regarded as a key molecule for the origin of life^7–9^. Therefore, determining whether the surface environments on early terrestrial planets fostered the production of H_2_CO is crucial for understanding prebiotic chemical evolution to the origin of life.

The evidence that supports the existence of liquid water have led many scientists to imagine a warm early Martian climate as it is on Earth^1,2^. Previous numerical studies have attempted to reproduce warm early Mars; however, 3-D global circulation model (GCM) studies have not been able to reproduce the continuous presence of liquid water on the surface with CO_2_-H_2_O atmospheres^10,11^. To reconcile this geomorphological evidence, episodic melting scenarios driven by the supply of reducing gases through volcanic outgassing or meteorite impacts have been proposed^12–15^.

Pinto et al.^16^ provided an estimate for the photochemical production of H_2_CO in the N_2_-dominated atmosphere of primitive Earth, with the number density of H_2_CO near the surface approximated to be ~ 10^8^ cm^−3^. A similar amount of H_2_CO production was also predicted for early Earth conditions by Harman et al.^17^. However, the production of H_2_CO in a CO_2_-dominated atmosphere on early Mars has not yet been thoroughly investigated. Understanding its production on early Mars can provide insights into the potential of life on the planet.

In this study, we investigated the production of H_2_CO in a thick CO_2_-dominated atmosphere containing H_2_ and CO on early Mars using a 1-D photochemical model. We then estimated ribose production using our simulation results and experimental data.

## Methods

### Model description

**Figure 1:**
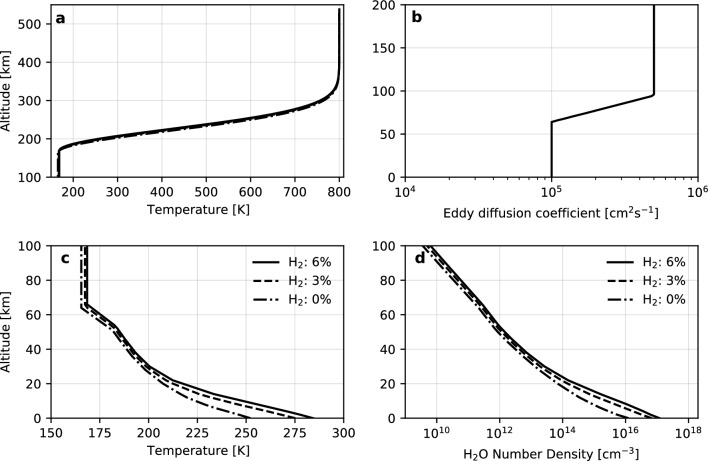
Background atmospheric conditions in early Mars. Temperature-altitude profile (**a** and **c**), Eddy diffusion coefficient profile (**b**), and H_2_O number density profile (**d**). The solid, dashed, and dash-dotted lines correspond to H_2_ 6%, 3%, and 0% conditions, respectively. The temperature and H_2_O number density profiles of the H_2_ 0% case in this figure are used to calculate the results for H2 0.1, 0.01, 0.001, and 0.0001% cases.

To calculate the atmospheric production of H_2_CO, we adapt a one-dimensional photochemical model, PROTEUS (Photochemical and RaiatiOn Transport model for Extensive USe), detailed by Nakamura et al.^18^, for early Martian conditions. This model has been successfully applied to other planetary atmospheres, such as the Jovian ionosphere^19^ and present-day Martian atmosphere^20^. It solves the continuity equations involving chemical reactions and vertical transport until the profiles of each species reach a steady state. We consider 63 chemical reactions (Supplementary Table S1 online) for 8 neutrals: CO_2_, CO, H_2_, H_2_O, O_2_, H_2_O_2_, O_3_, H_2_CO, 6 radicals: H, O, OH, HO_2_, O(^1^D), HCO, and an ion of CO_2_^+^ in a 2-bar CO_2_-dominated atmosphere. Though the atmospheric surface pressure of early Mars is still not well constrained, 3-D global circulation model studies suggest that a 2 bar CO_2_ atmosphere with a few percentages of reducing gas is required for a warm climate^14,15^. The exobase altitude is defined as a pressure level of 10^−9^ mbar. We utilize the H_2_O vapor number density and temperature profiles up to ~ 60 km (Fig. 1) from the 2-bar global mean results with an obliquity of 40° computed using a 3-D paleo-Mars global climate model^15,21^. The global climate model assumed three scenarios of an atmosphere containing 0, 3, and 6% H_2_. For the H_2_O density profiles above ~ 60 km, we assume the same mixing ratio up to the exobase, assuming the effect of cold trap. The sensitivity to the H_2_O vapor content in the atmosphere is discussed in Results section. For temperature, we assume isothermal up to the lower boundary of the thermosphere (6 × 10^−3^ Pa) considering radiative equilibrium and then extrapolate it into the thermosphere^22,23^. The exobase temperature is fixed at 800 K, corresponding to 10 × EUV at 3.8 Ga^24^. We adopt the solar spectrum from 3.8 Ga estimated by Claire et al.^25^. We use the updated H_2_O absorption cross section measured by Ranjan et al^26^. We assume the up-to-date absorption cross sections for all the species to the best of our knowledge. References for cross sections of all species at each wavelength are presented in Nakamura et al.^18^. The CO_2_^+^ concentration profile calculated by the ionosphere photochemistry model^27^ is fixed at the same pressure altitude as present-day Mars to represent the dissociation reaction of H_2_ with CO_2_^+^ to produce atomic H in the upper atmosphere, allowing us to calculate the escape flux of hydrogen^23^. Although the density of CO_2_^+^ in the early Martian atmosphere has uncertainty, its profile has little impact on the result, because the escape flux of hydrogen is limited by H_2_ diffusion from the lower atmosphere. We adopt eddy diffusion coefficient profiles using the typical formula adapted for other planets than Earth^28^. The vertical temperature, H_2_O number density, and eddy diffusion coefficient profiles are shown in Fig. 1. The temperature and water vapor profiles of the H_2_ 0% scenario shown in Fig. 1 are used to calculate the H_2_CO production under the conditions of 0.1, 0.01, 0.001, and 0.0001% H_2_.

For the upper boundary, we assume Jeans escape of H and H_2_ and fix the O escape rate at 2.6 × 10^8^ cm^−2^ s^−1^ corresponding to 10 × EUV conditions^24^. The O escape rate does not have a large impact on the results because it does not control O_2_ abundance in the lower atmosphere where H_2_CO is dominantly produced. Deposition velocities are applied to H_2_O_2_, HO_2_, H_2_CO, HCO, OH, O, and H^29,30^. The deposition velocities of each species are shown in Supplementary Table S2 online. We compute the dry deposition of H_2_CO by imposing the deposition velocity. This model also includes the rainout of H_2_CO throughout the atmosphere using the same parameterization as Hu et al^28^:

1 $${\text{k}}_{{\text{R}}} \left( {\text{z}} \right) = {\text{f}}_{{\text{R}}} \times \frac{{{\text{n}}_{{{\text{H}}_{2}{\text{O}}}} \left( {\text{z}} \right){\text{k}}_{{{\text{H}}_{2}{\text{O}}}} \left( {\text{z}} \right)}}{{55{\text{N}}_{{\text{A}}} \left[ {{\text{L}} \times 10^{ - 9} + \left( {{\text{H}}^{\prime } {\text{RT}}\left( {\text{z}} \right)} \right)^{ - 1} } \right]}},$$

where k_R_ is rainout frequency, f_R_ is a reduction factor which is an adjustable parameter to represent the reduction relative to Earth’s hydrological cycle, ${\text{n}}_{{\text{H}}_{2}{\text{O}}}$ is the number density of H_2_O, ${\text{k}}_{{\text{H}}_{2}{\text{O}}}$ is the precipitation rate assumed to be 2 × 10^–6^ s^−1^, N_A_ is Avogadro’s constant, L is the liquid water content assumed to be 1 g m^−3^, H′ is the effective Henry’s Law constant assumed to be 1.3 × 10^4^ M atm^−1^ taken from Giorgi & Chameides^31^, R is gas constant, and T is temperature. The rainout rate is then obtained by multiplying k_R_ by number density of H_2_CO. We assume f_R_ to be 1, assuming that early Mars had a hydrological cycle similar to Earth’s. Sensitivity to f_R_ is discussed in Results section. The boundary conditions for H_2_ and CO are fixed in the calculation of H_2_CO production in the results. We impose H_2_ outgassing and CO deposition velocity as a boundary condition to determine the possible range of H_2_ and CO mixing ratio in the following section.

### Background H_2_ and CO atmospheric conditions

Potential sources of H_2_ gas on early Mars include volcanic degassing^29^, meteorite impacts^32–34^, and serpentinization^35^. The upper limit of the H_2_ outgassing rate is estimated to be 8 × 10^11^ cm^−2^ s^−1^ considering the supplies from volcanism and serpentinization^29^. We computed H_2_ mixing ratios in a background 2-bar CO_2_ atmosphere for various H_2_ outgassing rates to investigate the plausibility of CO_2_ atmospheres enriched in H_2_, assuming a fixed CO deposition velocity of 10^−8^ cm s^−1^^29^ (see Supplementary Fig. S1 online). An H_2_ outgassing rate of ~ 5 × 10^11^ cm^−2^ s^−1^ yields a 5–6% H_2_ mixing ratio in a 2-bar CO_2_ atmosphere. This result is consistent with that of Batalha et al.^29^, in which an H_2_ outgassing rate of 8 × 10^11^ cm^−2^ s^−1^ yields a ~ 5% H_2_ mixing ratio in a 3-bar CO_2_ atmosphere. The minimum value of the H_2_ mixing ratio is ~ 1 × 10^–6^ when assuming the absence of H_2_ degassing. Furthermore, Chassefiere et al.^35^ suggested that serpentinization-derived CH_4_ trapped in the cryosphere could have been released into the atmosphere, producing a transient 1–2 bar CO_2_ atmosphere containing 10–20% H_2_ gas. Thus, a 6% H_2_ mixing ratio in a background 2-bar CO_2_ atmosphere is plausible. This study considers a range of H_2_ mixing ratios from 1 × 10^−6^ to 0.06.

The abundance of CO on early Mars is not well constrained. A dense and cold CO_2_ atmosphere is likely to enter the CO runaway state^23^. The CO and O liberated from CO_2_ photolysis no longer recombine because of the lack of odd hydrogen species that catalyze CO_2_ recombination. Moreover, because the equilibrium timescale of CO is relatively long, ranging from several million to several hundred million years^23^, it is insufficient to consider only a single steady state condition. Therefore, it is necessary to vary the CO mixing ratio as a parameter within a specific range.

To determine the possible CO range, we calculated the CO mixing ratios over a wide range of CO deposition velocities for 0, 3, and 6% H_2_ cases. Different temperatures and H_2_O profiles are used for each H_2_ case obtained from the GCM results^15^. We assume a free lower boundary condition for H_2_ in the 0% H_2_ case, whereas the number density is fixed for the 3 and 6% H_2_ conditions.

As a result, the 0% H_2_ case enters a CO runaway state with a low CO deposition velocity of < 10^−10^ cm s^−1^ (see Supplementary Fig. S2 online) as suggested by the previous photochemical model study of early Mars conditions^36^. Conversely, warmer atmospheres containing 3 or 6% H_2_ have a maximum CO mixing ratio of ~ 1% because sufficient H_2_O vapor promotes CO_2_ recombination. The CO deposition velocity on an abiotic ocean planet is estimated to be 10^−9^–10^−8^ cm s^−1^^37,38^; however, there is no known efficient process to remove CO at the surface without the ocean^28^. Considering that ancient Mars experienced episodic cold and warm climates^1^, a CO_2_ atmosphere would have been in a CO runaway state during the ice age^36^, while the atmosphere would have been stable with a minimum CO mixing ratio of 1% in warmer climates. Based on these results, the possible range of CO on early Mars should be from 1 to 50%.

## Results

### Photochemical production of formaldehyde

**Figure 2:**
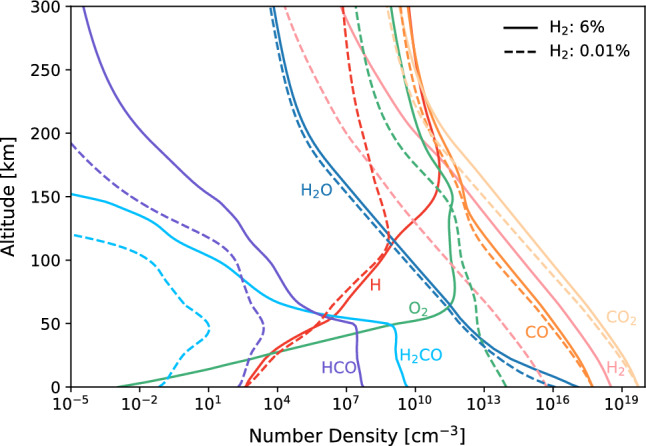
Number density profiles of main species under the 6% H_2_ (solid line) and 0.01% H_2_ (dashed line) conditions. CO is fixed at 1% in both cases.

The present 1-D photochemical model shows that H_2_CO forms at number densities of ~ 5 × 10^9^ and ~ 7 × 10^−2^ cm^−3^ near the surface under 6% H_2_ condition and 0.01% H_2_ condition, respectively (Fig. 2). Production in a 6% H_2_ atmosphere is substantial; approximately 50 times higher than that estimated for Earth's Hadean atmosphere^16^.

H_2_CO is predominantly formed through a radical–radical reaction of two HCO molecules^39^:

R1 $${\text{HCO}} + {\text{HCO}} \to {\text{H}}_{2} {\text{CO}} + {\text{CO}}{.}$$

The dominant production path of HCO is a three-body reaction of H and CO:

R2 $${\text{H}} + {\text{CO}} + {\text{M}} \to {\text{HCO}} + {\text{M,}}$$

where M represents the background gas, mainly CO_2_ in this model. In the early Martian atmosphere, H and CO in R2 are derived from H_2_O and CO_2_ photolysis, respectively. HCO is dominantly destroyed by a reaction with O_2_:

R3 $${\text{HCO}} + {\text{O}}_{2} \to {\text{HO}}_{2} + {\text{CO}}{.}$$

These reactions imply that the production of H_2_CO decreases with O_2_ because O_2_ reacts with HCO through R3, thereby reducing the rate of R1.

The H_2_CO production in the 6% H_2_ atmosphere is notably higher than that under lower H_2_ conditions. This results from the discrepancy in the O_2_ abundance in the lower atmosphere between these two conditions. There is a sharp decline in the O_2_ density below 60 km for 6% H_2_ (Fig. 2). The lack of O_2_ in the 6% H_2_ atmosphere decreased the reaction rate of HCO with O_2_ (R3). Consequently, a larger amount of HCO remains near the surface, ultimately increasing the amount of H_2_CO via reaction R1. Two mechanisms below contribute to the decrease in O_2_.

The first mechanism is driven by H atoms at altitudes greater than 100 km. The source of O_2_ near the surface is the downward transport of O_2_ liberated from CO_2_ photolysis at high altitudes. The significant difference between the 6% and 0.01% H_2_ cases is the number of H atoms at altitudes above 100 km. H_2_ is transported upward and photolyzed into H atoms by the solar UV flux, producing more H in the 6% H_2_ case. O_2_ reacts with H at high altitudes and is converted back into CO_2_ via the following reaction:

R4 $${\text{H}} + {\text{O}}_{2} + {\text{M}} \to {\text{HO}}_{2} + {\text{M}}$$

R5 $${\text{H}} + {\text{HO}}_{2} \to 2{\text{OH}}$$

R6 $${\text{CO}} + {\text{OH}} \to {\text{CO}}_{2} + {\text{H}}{.}$$

**Figure 3:**
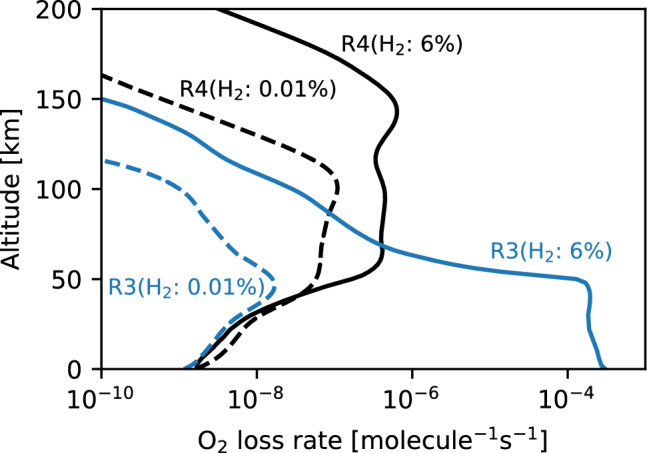
A comparison of O_2_ loss reaction rates per one O_2_ molecule (= reaction rate / O_2_ number density) under the 6% H_2_ (solid line) and 0.01% H_2_ (dashed line) conditions. The black and blue lines show the reaction rates of ${\text{H}} + {\text{O}}_{2} + {\text{M}} \to {\text{HO}}_{2} + {\text{M}}$ (R4) and ${\text{HCO}} + {\text{O}}_{2} \to {\text{HO}}_{2} + {\text{CO}}$ (R3), respectively.

**Figure 4:**
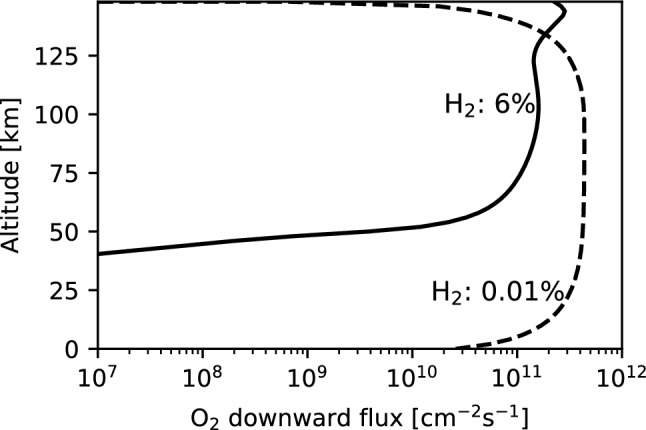
A comparison of O_2_ downward flux profiles under the 6% H_2_ (solid line) and 0.01% H_2_ (dashed line) conditions.

The altitude profiles of the reaction rate of R4 between the 6% and 0.01% H_2_ cases indicate that R4 shifted upward under the 6% H_2_ condition (Fig. 3). The loss of O_2_ by H atoms through R4 above ~ 150 km results in a decrease in O_2_ downward flux below ~ 135 km, as shown in Fig. 4.

The second mechanism is driven by the HCO catalytic cycle. In the present-day Martian atmosphere, odd hydrogen species act as catalysts to recombine CO and O into CO_2_ through the following cycle^40^:

R4 $${\text{H}} + {\text{O}}_{2} + {\text{M}} \to {\text{HO}}_{2} + {\text{M}}$$

R7 $${\text{O}} + {\text{HO}}_{2} \to {\text{OH}} + {\text{O}}_{2}$$

R6 $${\text{CO}} + {\text{OH}} \to {\text{CO}}_{2} + {\text{H}}$$

Net: $${\text{CO}} + {\text{O}} \to {\text{CO}}_{2}$$.

The net result is the recombination of CO_2_.

In a dense CO_2_ atmosphere, HCO catalytic reactions are responsible for converting H to HO_2_ in addition to R4:

R3 $${\text{HCO}} + {\text{O}}_{2} \to {\text{HO}}_{2} + {\text{CO}}$$

R2 $${\text{H}} + {\text{CO}} + {\text{M}} \to {\text{HCO}} + {\text{M}}$$

Net: $${\text{H}} + {\text{O}}_{2} \to {\text{HO}}_{2}$$.

This is because a larger amount of CO is formed in a denser atmosphere owing to the lack of amounts of odd hydrogen, while O_2_ is more abundant than CO in the present-day Martian atmosphere. The reaction rate profile of R3 in a 6% H_2_ atmosphere indicates that the HCO catalytic cycle dominates at ~ 50–60 km (Fig. 3), where the O_2_ density declines sharply (Fig. 2). This cycle significantly reduces O_2_ in the lower atmosphere below 50–60 km.

### Deposition of formaldehyde

A 6% H_2_ mixing ratio enables the presence of an ocean in a warm climate^15^. In a warm environment with an ocean and a CO deposition velocity of 10^−8^–10^−9^ cm s^−1^^37,38^, the CO mixing ratio is ~ 1%, as shown in the 3% and 6% H_2_ cases in Supplementary Fig. S2. Consequently, the deposition flux of H_2_CO into the surface liquid water is approximately 3 × 10^9^ cm^−2^ s^−1^ in a warm climate.

**Figure 5:**
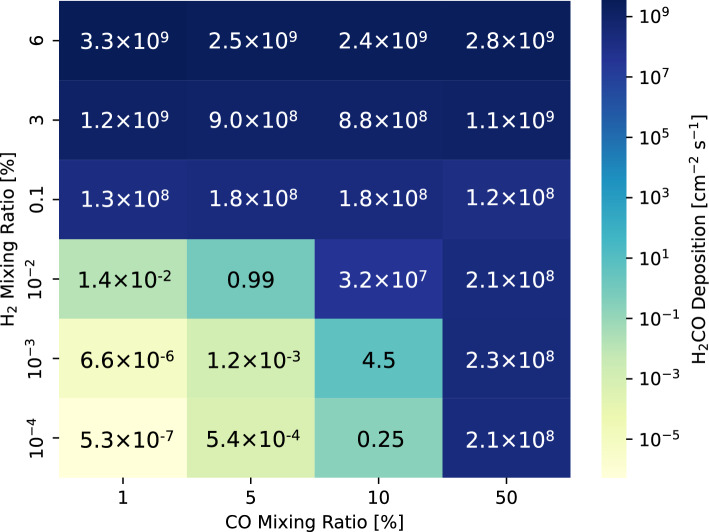
H_2_CO deposition flux as a function of CO and H_2_ mixing ratios assuming a 2-bar CO_2_ background atmosphere.

When the mixing ratio of H_2_ and CO decreases, the deposition of H_2_CO decreases. However, the H_2_CO decrease is not gradual, and there is a respective large drop with the decrease in H_2_ and CO. Large amounts of H_2_CO are deposited in an atmosphere containing either > 0.1% H_2_ or > 50% CO (Fig. 5).

The high-altitude H generates a large drop between 0.01 and 0.1% H_2_, as shown in Fig. 5. The large decrease between 10 and 50% CO is associated with the HCO catalytic cycle (Fig. 5). Increasing the CO produces more HCO (R2), thereby removing O_2_ in the lower atmosphere (R3). In an atmosphere containing 10% CO, a steady state is reached with more O_2_ and less HCO. Conversely, the atmosphere containing 50% CO reaches a steady state with less O_2_ and more HCO. Therefore, in an atmosphere with H_2_ < 0.01%, more H_2_CO is produced with 50% CO.

### Formaldehyde deposition in more abundant H_2_ and H_2_O conditions

**Figure 6:**
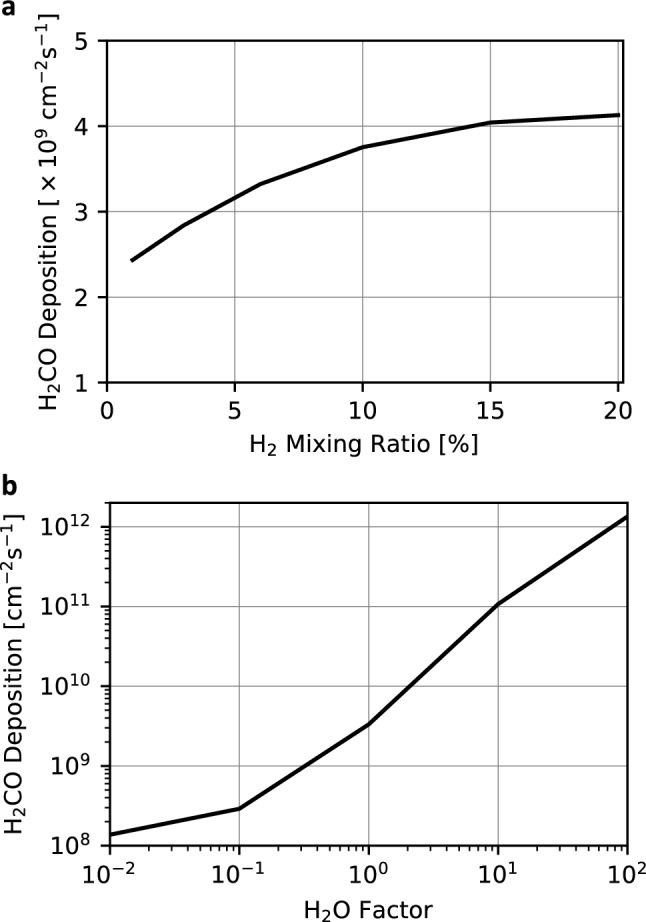
H_2_CO deposition fluxes as a function of the H_2_ mixing ratio (**a**) and H_2_O factor (**b**). The temperature profile of the 6% H_2_ case is used for both calculations. The bottom panel (**b**) shows H_2_CO deposition fluxes obtained by multiplying the H_2_O profile of the 6% H_2_ case by a factor ranging from 0.01 to 100.

We first assessed the impact of a higher H_2_ mixing ratio than 6% on H_2_CO production, with the CO mixing ratio fixed at 1% and H_2_O and temperature profiles from the 6% H_2_ GCM results^15^. This assumption may not represent early Martian conditions, but it allows the estimation of the upper limit of H_2_CO production in a CO_2_-dominated atmosphere on early Mars and provides insights into an exoplanet analog for a CO_2_-dominated atmosphere enriched with H_2_. As shown in Fig. 6, increasing the H_2_ mixing ratio from 1 to 20% increased the H_2_CO deposition slightly. However, the deposition flux does not exceed 5 × 10^9^ cm^−2^ s^−1^. This result implies that H_2_CO production under early Martian atmospheric conditions would be close to its maximum in H_2_-rich CO_2_-dominated atmospheres.

Subsequently, we multiplied the H_2_O profile of the 6% H_2_ case by a factor ranging from 0.01 to 100 while maintaining the H_2_ and CO mixing ratios at 6% and 1%, respectively, with the temperature profile of the 6% H_2_ case. This approach accounts for spatiotemporal variations in water vapor content on a global scale. H_2_O vapor changes by ~ 10 times over the global scale in the global climate model results^21^. Considering the seasonal difference, changes by ~ 2 orders of magnitude are reasonable for early Mars conditions. As an upper and lower limit, we changed the amount of H_2_O vapor by 4 orders of magnitude. This parameter survey may also be useful for exoplanets’ environment. A higher water vapor content significantly increases H_2_CO production (Fig. 6b). The H_2_CO deposition flux increases up to ~ 1 × 10^12^ cm^−2^ s^−1^ with a 100 times higher H_2_O density. Under conditions of abundant H_2_O, additional H atoms are generated through H_2_O photolysis, resulting in increased HCO formation through R2 and, consequently, greater H_2_CO production through R1. In addition, an increase in precipitation also contributes to the increased rainout rate of H_2_CO. This indicates that in a warm climate with 6% H_2_, the limiting factor for H_2_CO production is not H_2_ but the abundance of H_2_O. The timescale for the change in H_2_CO formation due to variations in water vapor content is a few years in our calculation. This result suggests that H_2_CO production on early Mars exhibited local variations depending on the availability of water vapor. However, further studies are needed to clarify this effect, as horizontal transport may mitigate differences in H_2_CO abundance.

We also investigated the effect of the reduction factor f_R_ in Eq. (1) on H_2_CO deposition flux. The calculated H_2_CO deposition fluxes with a reduction factor of 0.1, 0.5, and 1 are shown in Supplementary Fig. S4. In this calculation, H_2_ and CO mixing ratios are fixed at 6% and 1%, respectively, with temperature and H_2_O profiles of the 6% H_2_ case. When f_R_ is set to 0.1, it is reduced to 8 × 10^8^ cm^−2^ s^−1^, approximately 1/4 of the value when f_R_ is 1. Although it is not yet constrained, GCM results suggest that globally averaged precipitation on early Mars may have been 10 times smaller than on Earth^15^. Further modeling studies combining photochemistry with GCM would be helpful for a more accurate estimation of the rainout rate.

## Discussion

### Formation of formaldehyde throughout Mars’ history

**Figure 7:**
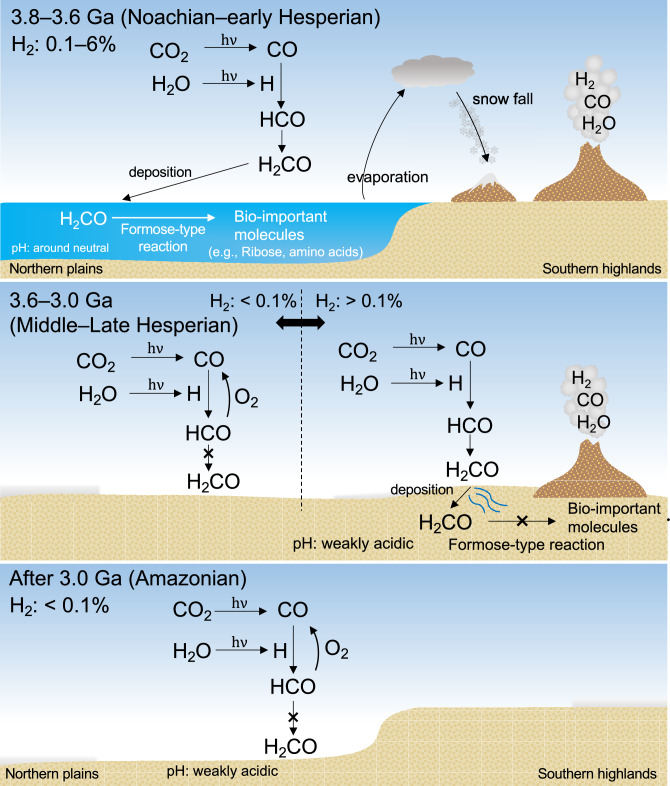
Scenario for the atmospheric H_2_CO production at ca. 3.8–3.6 Ga (top panel), ca. 3.5–3.0 Ga (middle panel), and after ca. 3.0 Ga (bottom panel). In the Noachian and early Hesperian periods (3.8–3.6 Ga), the synthesized H_2_CO in the atmosphere was deposited into the ocean, forming bio-important molecules, such as ribose. In the middle and late Hesperian (3.6–3.0 Ga), H_2_CO was sporadically formed. Even in the period when H_2_CO was abundantly formed, subsequent formose reaction does not proceed due to the acidic condition of the water. From the Amazonian to the present (after 3.0 Ga), the production of H_2_CO is deficient, as in the case with H_2_ < 0.1% in the middle and late Hesperian.

The deposition rate of H_2_CO reaches the order of 10^8^ or 10^9^ cm^−2^ s^−1^ under conditions where the mixing ratio of H_2_ is higher than 0.1%, regardless of the CO mixing ratio (Fig. 5). The H_2_ mixing ratio of 0.1% is equivalent to ~ 10^10^ cm^−2^ s^−1^ of H_2_ outgassing rate in a steady state (Supplementary Fig. S1). This rate is comparable to the estimated H_2_ degassing rates on present-day Earth, which has an upper mantle of quartz-fayalite-magnetite (QFM) oxidation buffer^29,41^. The H_2_ degassing rate on early Mars is unclear. However, Martian meteorites suggest that the mantle was more reduced than the Earth’s upper mantle, with oxygen fugacity around the iron-wüstite (IW) buffer^42,43^. Given that the oxygen fugacity of the Martian upper mantle was buffered near IW + 1, the H_2_ degassing rate is estimated to be ~ 10^11^ cm^−2^ s^−1^^29,41^. This suggests that the large atmospheric production of H_2_CO continued during past periods of active volcanic degassing, regardless of the CO mixing ratio (Fig. 7). An increase in the H_2_ mixing ratio from 0.1 to 6% increases the H_2_CO deposition rate by approximately 10 times the global mean value (Fig. 5). This increase was mainly due to the increase in the number density of H_2_O by 10 times due to the warming effect of increasing H_2_ (Fig. 1d). The number density of H_2_O in the atmosphere also differs (e.g., 10 times) depending on the local availability of H_2_O^15^. This difference could provide local H_2_CO deposition rates on Mars that are several tens of times higher and lower than the global mean value (Fig. 6b).

Volcanic degassing would have decreased from the late Hesperian to the Amazonian, decreasing the mixing ratio of H_2_ and H_2_O in the atmosphere^42^. A decrease in the H_2_ mixing ratio to below 0.1% dramatically decreased the H_2_CO deposition rate by a factor of 10^−10^ (Fig. 5). Thus, the late Hesperian to early Amazonian was a transitional period from a high to a meager H_2_CO deposition rate (Fig. 7).

### Formation of organic compounds in the ocean

The continuous conversion of CO_2_ and CO into highly soluble H_2_CO in the early Martian atmosphere may have transferred carbon from the atmosphere to the ocean. Another mechanism that converts atmospheric carbon into H_2_CO involves iron-rich asteroids/meteorites^44^. Such impacts might have formed H_2_CO, both locally and temporally. The overall impact-induced production would have been smaller than the continuous global production of H_2_CO in the atmosphere through the photochemical reactions presented in this study (see Supplementary text online). Another source of H_2_CO discussed previously is the oxidation of CH_4_ with iron oxide, which was proposed to explain the tentative detection of H_2_CO on present-day Mars^45^.

H_2_CO is highly reactive. Carbon transferred from the atmosphere as H_2_CO could further be converted into various organic compounds. One of the most well-known reactions is the formose reaction, in which formaldehyde oligomerizes to form various sugar molecules in alkaline solutions^7^. A recent study found that this type of reaction can form sugars, including ribose, even in neutral solutions^46^. To estimate ribose production in the early Martian ocean, we make the following assumptions: one-third of the surface area was covered by an ocean as suggested by the analysis of the distribution of delta and valleys^47^, the seawater pH at ~ 3.8 Ga in early Mars was near-neutral, as the late Noachian marked a transition period from alkaline to acidic water pH^48^, early Mars had a hydrological cycle similar to Earth’s (f_R_ = 1), the atmosphere contained the same fraction of glycolaldehyde as an estimated atmosphere of the early Earth^17^, and the conversion rate of H_2_CO into ribose was ~ 3.5 × 10^−6^ mol_Rib_ mol_FA_^−1^ estimated by the formose-reaction experiment^46^. By combining the calculated H_2_CO deposition flux of 3 × 10^9^ cm^−2^ s^−1^ (= 1 × 10^21^ m^−2^ yr^−1^), the annual ribose production in the ocean P_rib_ is estimated to be 4 × 10^4^ kg yr^−1^ as follows:

2 $${\text{P}}_{{{\text{rib}}}} = \frac{{{\text{f}}_{{{\text{H}}_{2} {\text{CO}}}} {\text{S}}_{{{\text{sea}}}} {\text{Y}}_{{{\text{rib}}}} {\text{M}}_{{{\text{rib}}}} }}{{{\text{N}}_{{\text{A}}} }},$$

where ${\text{f}}_{{\text{H}}_{2}{\text{CO}}}$ is the deposition flux of H_2_CO (m^−2^ yr^−1^), S_sea_ is the area covered by the ocean (m^2^), Y_rib_ is the conversion rate of H_2_CO into ribose (mol mol^−1^), M_rib_ is the molar mass of ribose (kg mol^−1^), and N_A_ is Avogadro’s constant. When f_R_ is 0.1, the annual ribose production is estimated to be 1 × 10^4^ kg yr^−1^. These suggest that bio-important sugars including ribose might have been continuously formed in water bodies on the surface. In this assumption, we disregarded several H_2_CO consumption processes in seawater, including photolysis, hydrolysis, and reactions with other reactive molecules such as ammonia^3,49^. It is unclear whether ammonia was present on early Mars, but it may have transiently been present in the atmosphere or in water on early Mars, potentially due to processes, such as episodic volcanic degassing or impact degassing. Previous studies on early Earth indicated that reducing gases, including ammonia, could be generated through impact events^50,51^. This process may have analogously occurred on early Mars. In the presence of ammonia, the formose reaction forms various nitrogen containing organic matter including proteinogenic amino acids^5^. Nitrogen containing organic matter has been found in a Martian meteorite^52^. The formose reaction also forms refractory organic matter, similar to cometary and meteoritic insoluble organic matter^6^. The photochemical H_2_CO calculated in this study and its following formose reaction may be related to the origin of refractory and non-refractory organic matter found in the 3.5-billion-year-old lacustrine mudstones of Mars^53^ and Noachian carbonates in a Martian meteorite^52^. However, it is still difficult to distinguish whether this organic matter was derived from the photochemical H_2_CO. One way to distinguish them is to compare their carbon isotope compositions. The carbon isotopic analysis onboard the Curiosity rover detected an anomalously depleted ^13^C in organic matter^54^. The deposition of photochemical H_2_CO which experienced CO_2_ photolysis-driven carbon isotope fractionation might explain this depletion^55^. Our future work is to include carbon isotope fractionation in the model and compare it with the isotope observation data. Significant H_2_CO synthesis on the warm Noachian Mars with liquid surface water allowed for the formation of sugars and amino acids (Fig. 7). The formation of H_2_CO would have sporadically continued on Hesperian to early Amazonian Mars, but the transition to an ice-covered and acidic surface environment on Hesperian Mars dramatically decreased the possibility of the formation of the building blocks of life, because the production of sugars and amino acids through the formose-type reaction substantially decreased in acidic water^46,48,56,57^. Therefore, the time period suitable for the formation of bio-important molecules on early Mars might be limited to the Noachian and potentially early Hesperian Mars, the warm climate era before the pH of the surface liquid water became acidic.

Secondary concentration processes are essential for synthesizing bio-important molecules on planetary surfaces. Early Mars may have experienced episodic warm and cold climate periods^33^. During the transition from warm to cold periods, a large amount of oceanic water would have been stored as snow on land; thus, oceanic water would have become concentrated. Such evaporative environments might further promote chemical evolution to form biopolymers, such as proteins and RNAs because the primary reaction that forms these molecules is dehydration reactions^58,59^. These reactions may have been promoted by carbonate and borate, which have been shown to be present on Mars^60–64^. Future studies considering topography and secondary concentration processes are important to elucidate the possibility of RNA synthesis on early Mars.

## Conclusions

Formaldehyde (H_2_CO) is a crucial organic matter in the formation of bioorganic molecules such as amino acids and ribose. We investigated the atmospheric production of H_2_CO on early Mars using a one-dimensional photochemical model. We assume a 2-bar background CO_2_-dominated atmosphere with various concentrations of H_2_ and CO while adopting temperature and H_2_O profiles from a 3-D paleo-Mars global climate model. Our results show that a larger amount of H_2_ leads to a more significant production of H_2_CO owing to the reduction in O_2_ abundance in the lower atmosphere. Two mechanisms cause this O_2_ reduction: (1) the chemical reaction with H atoms at high altitudes above 100 km, resulting in a decrease in O_2_ downward flux, and (2) the HCO catalytic cycle at ~ 50–60 km reducing O_2_ in the lower atmosphere below 60 km. In a warm climate, the number density of H_2_CO is ~ 5 × 10^9^ cm^−3^ near the surface, and its deposition into the ocean is 3 × 10^9^ cm^−2^ s^−1^ assuming that early Mars had a hydrological cycle similar to Earth’s. The sensitivity analysis of water vapor implies that H_2_CO production could have varied locally in correlation with the abundance of water vapor. Our results suggest that a continuous supply of H_2_CO could be used to form various organic compounds, including life's building blocks, such as amino acids and sugars. This photochemically produced H_2_CO could be a possible origin for the organic matter observed on the Martian surface. Given the previously reported conversion rate from H_2_CO to ribose, the calculated H_2_CO deposition flux suggests a continuous supply of bio-important sugars on Noachian and early Hesperian Mars.

### Supplementary Information

Supplementary Information.

## Data Availability

The data of the simulation results is available at figshare repository: https://doi.org/10.6084/m9.figshare.24032064.
