# Supplementary material for: Atmospheric formaldehyde production on early Mars leading to a potential formation of bio-important molecules
Source: Sci Rep. 2024 Feb 9;14:2397. doi: 10.1038/s41598-024-52718-9 (PMC10858170; doi:10.1038/s41598-024-52718-9)
Supplement: Supplementary file 1 — Supplementary Information. [file 41598_2024_52718_MOESM1_ESM.docx]

**Supplementary Information**

**Atmospheric formaldehyde production on early Mars leading to a potential formation of bio-important molecules**

Shungo Koyama^1*^, Arihiro Kamada^1^, Yoshihiro Furukawa^1^, Naoki Terada^1^, Yuki Nakamura^2^, Tatsuya Yoshida^1^, Takeshi Kuroda^1,3^, and Ann Carine Vandaele^4^

^1^*Graduate School of Science, Tohoku University, Sendai, Japan*

*^2^Graduate School of Science, The University of Tokyo, Tokyo, Japan*

^3^*Division for the Establishment of Frontier Sciences of Organization for Advanced Studies, Tohoku University, Sendai, Japan*

*^4^Royal Belgian Institute for Space Aeronomy, BIRA-IASB, Brussels, Belgium*

*Corresponding author:

Shungo Koyama

Graduate School of Science

Tohoku University

Sendai, Miyagi 980-8578, Japan

Email: [koyama.shungo.q5@dc.tohoku.ac.jp](mailto:koyama.shungo.q5@dc.tohoku.ac.jp)

**1 Estimation of impact-induced H_2_CO production**

The impact-induced H_2_CO production on early Earth was estimated to be ~ 9 × 10^16^ mol in total or ~ 9 × 10^8^ mol yr^-1^ during the Late Heavy Bombardment (LHB)^1^. We scale from the impact-induced H_2_CO production on early Earth to that on early Mars. We account for the Martian smaller collision cross section $\sigma_{\mathrm{col}},$ which can be written by

| $\sigma_{col}=\pi R^{2}(1+\frac{v_{esc}^{2}}{v_{\infty}^{2}})$*,* | (S1) |
| --- | --- |

where R is the radius of the planet, v_esc_ is the escape velocity of the planet, and $v_{\infty}$ is the velocity of meteorite to the planet that is assumed to be 12 km s^-1^ as the previous earth’s calculation^2^. The ratio of the cross section of Mars to that of Earth is 0.18, thus the impact-induced H_2_CO production on early Mars is estimated to be 9 × 10^8^ × 0.18 = ~ 2 × 10^8^ mol yr^-1^. The photochemical production of H_2_CO presented in this study is 3 × 10^9^ cm^−2^ s^−1^ or 2 × 10^11^ mol yr^-1^ on Mars, which is larger than the estimated impact-induced production by about three orders of magnitude. The overall impact-induced production of H_2_CO would have been smaller than the continuous global photochemical production calculated in this study. Please note that this is a simple scaling estimation, and a detailed numerical simulation is required for a more realistic estimation.

**Fig. S1.** The H_2_ mixing ratio as a function of H_2_ outgassing flux.

**Fig. S2.** CO mixing ratio as a function of CO deposition velocity. The solid, dashed, and dash-dotted lines show the results of H_2_ 6%, 3%, and 0% conditions. Note that the 0% H_2_ case does not impose the constant H_2_ mixing ratio of 0%. Instead, for the 0% H_2_ case, we use the temperature and water vapor profiles derived from the GCM calculation under 0% H_2_ (see Fig.　1) and assume no H_2_ degassing at the surface.

**Fig. S3.** Number density profiles of all species. H_2_ is fixed at 6% and 0.01% in the upper and lower panel, respectively. CO is fixed at 1% in both cases.

Fig. S4. H_2_CO deposition fluxes as a function of reduction factors. A reduction factor is an adjustable parameter in the parameterization of the rainout rate, representing the reduction relative to Earth’s hydrological cycle^3^. In this calculation, H_2_ and CO mixing ratios are fixed at 6% and 1%, respectively, with temperature and H_2_O profiles of the 6% H_2_ case.

**Table S1**

Chemical reactions and column-integrated rates

| # | Reaction | | | Rate coefficient | Column rate [cm^-2^ s^-1^] | | Reference |
| --- | --- | --- | --- | --- | --- | --- | --- |
|  | | | | | CO: 1%  H_2_: 6% | CO: 1%  H_2_: 0.01% |  |
| 1 | CO₂ + hν | → | CO + O | Photodissociation | 4.35E+12 | 4.37E+12 |  |
| 2 | CO₂ + hν | → | CO + O(¹D) | Photodissociation | 4.85E+11 | 5.13E+11 |  |
| 3 | H₂O + hν | → | H + OH | Photodissociation | 3.20E+10 | 5.96E+09 |  |
| 4 | H₂O + hν | → | H₂ + O(¹D) | Photodissociation | 5.38E+06 | 1.22E+07 |  |
| 5 | O₃ + hν | → | O₂ + O | Photodissociation | 2.15E+10 | 1.26E+12 |  |
| 6 | O₃ + hν | → | O₂ + O(¹D) | Photodissociation | 1.22E+11 | 7.09E+12 |  |
| 7 | O₂ + hν | → | O + O | Photodissociation | 8.70E+10 | 1.04E+11 |  |
| 8 | O₂ + hν | → | O + O(¹D) | Photodissociation | 2.06E+11 | 1.52E+11 |  |
| 9 | H₂ + hν | → | H + H | Photodissociation | 1.69E+07 | 2.68E+05 |  |
| 10 | OH + hν | → | O + H | Photodissociation | 3.13E+06 | 1.56E+05 |  |
| 11 | OH + hν | → | O(¹D) + H | Photodissociation | 2.38E+06 | 1.02E+04 |  |
| 12 | HO₂ + hν | → | OH + O | Photodissociation | 1.11E+10 | 3.47E+10 |  |
| 13 | H₂O₂ + hν | → | OH + OH | Photodissociation | 3.85E+10 | 3.81E+11 |  |
| 14 | H₂O₂ + hν | → | HO₂ + H | Photodissociation | 3.79E+09 | 3.40E+10 |  |
| 15 | H₂O₂ + hν | → | H₂O + O(¹D) | Photodissociation | 0.00E+00 | 0.00E+00 |  |
| 16 | H₂CO + hν | → | HCO + H | Photodissociation | 1.27E+11 | 2.72E+02 |  |
| 17 | H₂CO + hν | → | CO + H₂ | Photodissociation | 1.39E+11 | 2.96E+02 |  |
| 18 | HCO + hν | → | H + CO | Photodissociation | 1.48E+10 | 7.69E+05 |  |
| 19 | O + O + M | → | O₂ + M | 5.4 × 10⁻³³ × (300/Tn)³·²⁵ | 1.12E+11 | 4.89E+11 | a |
| 20 | O + O₂ + CO₂ | → | O₃ + CO₂ | 1.5 × 10⁻³³ × (300/Tn)²·⁴ | 2.60E+11 | 8.72E+12 | a |
| 21 | O + O₃ | → | O₂ + O₂ | 8.0 × 10⁻¹² × exp(-2060/Tn) | 3.73E+06 | 2.39E+08 | a |
| 22 | O + CO + M | → | CO₂ + M | 2.2 × 10⁻³³ × exp(-1780/Tn) | 1.98E+12 | 2.05E+12 | a |
| 23 | O(¹D) + O₂ | → | O + O₂ | 3.2 × 10⁻¹¹ × exp(70/Tn) | 1.44E+10 | 3.99E+09 | a |
| 24 | O(¹D) + O₃ | → | O₂ + O₂ | 1.2 × 10⁻¹⁰ | 2.00E+02 | 8.51E+04 | a |
| 25 | O(¹D) + O₃ | → | O + O + O₂ | 1.2 × 10⁻¹⁰ | 2.00E+02 | 8.51E+04 | a |
| 26 | O(¹D) + H₂ | → | H + OH | 1.2 × 10⁻¹⁰ | 4.11E+10 | 6.93E+08 | a |
| 27 | O(¹D) + CO₂ | → | O + CO₂ | 7.5 × 10⁻¹¹ × exp(115/Tn) | 7.71E+11 | 7.75E+12 | a |
| 28 | O(¹D) + H₂O | → | OH + OH | 1.63 × 10⁻¹⁰ × exp(60/Tn) | 1.20E+06 | 5.34E+07 | a |
| 29 | H₂ + O | → | OH + H | 6.34 × 10⁻¹² × exp(-4000/Tn) | 2.29E+11 | 5.01E+08 | a |
| 30 | OH + H₂ | → | H₂O + H | 9.01 × 10⁻¹³ × exp(-1526/Tn) | 3.06E+10 | 9.23E+07 | a |
| 31 | H + H + CO₂ | → | H₂ + CO₂ | 1.6 × 10⁻³² × (298/Tn)²·²⁷ | 3.06E+10 | 3.57E+07 | a |
| 32 | H + OH + CO₂ | → | H₂O + CO₂ | 1.292 × 10⁻³⁰ × (300/Tn)²· | 2.72E+07 | 3.64E+05 | a |
| 33 | H + HO₂ | → | OH + OH | 7.2 × 10⁻¹¹ | 5.57E+11 | 1.85E+10 | a |
| 34 | H + HO₂ | → | H₂O + O(¹D) | 1.6 × 10⁻¹² | 1.24E+10 | 4.11E+08 | a |
| 35 | H + HO₂ | → | H₂ + O₂ | 3.45 × 10⁻¹² | 2.67E+10 | 8.86E+08 | a |
| 36 | H + H₂O₂ | → | HO₂ + H₂ | 2.8 × 10⁻¹² × exp(-1890/Tn) | 1.83E+06 | 2.91E+05 | a |
| 37 | H + H₂O₂ | → | H₂O + OH | 1.7 × 10⁻¹¹ × exp(-1800/Tn) | 1.86E+07 | 2.83E+06 | a |
| 38 | H + O₂ + M | → | HO₂ + M | k₀ = 8.8 × 10⁻³² × (300/Tn)¹·³ | 1.73E+12 | 2.15E+12 | a |
|  |  |  |  | k∞ = 7.5 × 10⁻¹¹ × (300/Tn)⁻⁰·² |  |  |  |
| 39 | H + O₃ | → | OH + O₂ | 1.4 × 10⁻¹⁰ × exp(-470/Tn) | 1.17E+11 | 3.68E+11 | a |
| 40 | O + OH | → | O₂ + H | 1.8 × 10⁻¹¹ × exp(180/Tn) | 8.00E+11 | 2.07E+11 | a |
| 41 | O + HO₂ | → | OH + O₂ | 3.0 × 10⁻¹¹ × exp(200/Tn) | 1.90E+12 | 1.82E+12 | a |
| 42 | O + H₂O₂ | → | OH + HO₂ | 1.4 × 10⁻¹² × exp(-2000/Tn) | 7.02E+06 | 1.69E+07 | a |
| 43 | OH + OH | → | H₂O + O | 1.8 × 10⁻¹² | 4.55E+06 | 1.64E+04 | a |
| 44 | OH + OH + M | → | H₂O₂ + M | k₀ = 8.97 × 10⁻³¹ × (300/Tn)¹· | 5.46E+02 | 3.21E+01 | a |
|  |  |  |  | k∞ = 2.6 × 10⁻¹¹ |  |  |  |
| 45 | OH + O₃ | → | HO₂ + O₂ | 1.7 × 10⁻¹² × exp(-940/Tn) | 7.19E+03 | 8.33E+04 | a |
| 46 | OH + HO₂ | → | H₂O + O₂ | 4.8 × 10⁻¹¹ × exp(250/Tn) | 1.01E+08 | 3.03E+07 | a |
| 47 | OH + H₂O₂ | → | H₂O + HO₂ | 1.8 × 10⁻¹² | 1.15E+07 | 6.20E+06 | a |
| 48 | HO₂ + O₃ | → | OH + O₂ + O₂ | 1.0 × 10⁻¹⁴ × exp(-490/Tn) | 1.54E+06 | 4.12E+08 | a |
| 49 | HO₂ + HO₂ | → | H₂O₂ + O₂ | 3.0 × 10⁻¹³ × exp(460/Tn) | 3.80E+10 | 1.61E+11 | a |
| 50 | HO₂ + HO₂ + M | → | H₂O₂ + O₂ + M | 4.2 × 10⁻³³ × exp(920/Tn) | 4.31E+09 | 2.59E+11 | a |
| 51 | CO + OH + M | → | CO₂ + H + M | k₀M = 1.5 × 10⁻¹³ × (300/Tn)⁻⁰·⁶ | 2.76E+12 | 2.83E+12 | a |
|  |  |  |  | k∞M = 2.1 × 10⁹ × (300/Tn)⁻⁶·¹ |  |  |  |
| 52 | CO₂⁺ + H₂ | → | H + H | 8.7 × 10⁻¹⁰ | 4.71E+11 | 5.80E+08 | a |
| 53 | H₂CO + O(¹D) | → | HCO + OH | 4.6 × 10⁻¹⁰ | 4.49E-01 | 1.25E-04 | b |
| 54 | H + CO + M | → | HCO + M | k₀ = 1.4 × 10⁻³⁴ × exp(-100/Tn) | 1.44E+12 | 5.40E+11 | c |
|  |  |  |  | k∞ = 1.96 × 10⁻¹³ × exp(-1366/Tn) |  |  |  |
| 55 | H + HCO | → | H₂ + CO | 1.8 × 10⁻¹⁰ | 4.50E+09 | 1.61E+07 | c |
| 56 | HCO + HCO | → | H₂CO + CO | 4.5 × 10⁻¹¹ | 2.69E+11 | 5.77E+02 | c |
| 57 | OH + HCO | → | H₂O + CO | 1.7 × 10⁻¹⁰ | 7.34E+05 | 4.42E+03 | c |
| 58 | O + HCO | → | H + CO₂ | 5.0 × 10⁻¹¹ | 7.70E+10 | 1.68E+08 | c |
| 59 | O + HCO | → | OH + CO | 5.0 × 10⁻¹¹ | 7.70E+10 | 1.68E+08 | c |
| 60 | H₂CO + H | → | H₂ + HCO | 2.1 × 10⁻¹⁶ × (Tn)^(1.62) × exp(-1090/Tn) | 1.55E+06 | 4.09E-02 | c |
| 61 | H₂CO + OH | → | H₂O + HCO | 8.2 × 10⁻¹² × exp(40/Tn) | 1.18E+06 | 1.76E-01 | c |
| 62 | O + H₂CO | → | OH + HCO | 3.4 × 10⁻¹¹ × exp(-1600/Tn) | 4.97E+08 | 8.41E+00 | c |
| 63 | HCO + O₂ | → | HO₂ + CO | 5.6 × 10⁻¹² × (Tn/298)⁻⁰·⁴ | 8.54E+11 | 5.40E+11 | d |

Notes. In the reference list, a, b, c, and d denote Chaffin et al.^4^, Pearce et al.^5^, Tian et al.^6^, and Batalha et al.^7^, respectively.

**Table S2**

Deposition Velocities for chemical species

| Species | Deposition (cm s^-1^) | Reference |
| --- | --- | --- |
| H_2_O_2_ | 0.2 | Batalha et al. (2015) |
| HO_2_ | 1 | Batalha et al. (2015) |
| H_2_CO | 0.1 | Pearce et al. (2022) |
| HCO | 1 | Batalha et al. (2015) |
| OH | 1 | Batalha et al. (2015) |
| O | 1 | Batalha et al. (2015) |
| H | 1 | Batalha et al. (2015) |

Notes. We primarily refer to Batalha et al.^7^. The H_2_CO dry deposition is calculated using the the deposition velocity in Pearce et al.^8^.

**Reference**

1. Masuda, S., Furukawa, Y., Kobayashi, T. Experimental Investigation of the Formation of Formaldehyde by Hadean and Noachian Impacts. *Astrobiology* **21**, 413-420 (2021).
2. Chyba, C. F. Terrestrial Mantle Siderophiles and the Lunar Impact Record. *Icarus* **92**, 217-233 (1991).
3. Hu, R., Seager S., Bains, W., Photochemistry in terrestrial exoplanet atmospheres. I. Photochemistry model and benchmark cases. *Astrophys. J.* **761**, 166 (2012).
4. Chaffin, M. S., Deighan, J., Schneider, N. M., & Stewart, A. I. F. Elevated atmospheric escape of atomic hydrogen from Mars induced by high-altitude water. *Nat. Geosci.* 10, 174-178 (2017).
5. Pearce, B. K., Ayers, P. W., & Pudritz, R. E. Crahcn-o: A consistent reduced atmospheric hybrid chemical network oxygen extension for hydrogen cyanide and formaldehyde chemistry in CO2-, N2-, H2O-, CH4-, and H2-dominated atmospheres. *J. Phys. Chem. A.* ***124*** 8594-8606 (2020).
6. Tian, F., Kasting, J.F., Zahnle, K., Revisiting HCN formation in Earth's early atmosphere. *Earth Planet. Sci. Lett.* **308**, 417-423 (2011).
7. Batalha, N., Domagal-Goldman, S. D., Ramirez, R., & Kasting, J. F. Testing the early Mars H2–CO2 greenhouse hypothesis with a 1-D photochemical model. *Icarus* **258**, 337-349 (2015).
8. Pearce., B.K. et al. Toward RNA life on early Earth: From atmospheric HCN to biomolecule production in warm little ponds. *Astrophys. J.* **932**, 9 (2022).
